# Advances in transdermal siRNAs delivery: A review of current research progress

**DOI:** 10.1016/j.ncrna.2023.05.008

**Published:** 2023-05-26

**Authors:** Albert Sufianov, Aferin Beilerli, Valentin Kudriashov, Tatiana Ilyasova, Bu Wenjie, Ozal Beylerli

**Affiliations:** a Educational and Scientific Institute of Neurosurgery, Рeoples’ Friendship University of Russia (RUDN University), Moscow, Russia; b Department of Neurosurgery, Sechenov First Moscow State Medical University (Sechenov University), Moscow, Russia; c Department of Obstetrics and Gynecology, Tyumen State Medical University, 54 Odesskaya Street, 625023, Tyumen, Russia; d Gastric Cancer Center, West China Hospital of Sichuan University, China; e Department of Internal Diseases, Bashkir State Medical University, Ufa, Republic of Bashkortostan, 450008, Russia; f Department of Pharmacology, College of Pharmacy, Harbin Medical University, 157 Baojian Rd, Nangang, Harbin, Heilongjiang, 150088, China

**Keywords:** RNA interference, Small interfering RNA, Delivery vector, Skin diseases, Lipidosome, Polymers, Nanoparticles

## Abstract

Small interfering RNA (siRNAs) is a double-stranded RNA molecule which can hybridize with a specific mRNA sequence and block the translation of numerous genes to regulate endogenous genes and to defend the genome from invasive nucleic acids. The use of siRNAs has been studied as a treatment option for various skin conditions. One of the main obstacles in the dermal or transdermal delivery of this compound is low skin permeability, and application is limited by its negative charge, high polarity, susceptibility to degradation by nucleases, and difficulty in penetrating the skin barrier. Effective delivery of therapeutic biomolecules to their target is a challenging issue, which can be solved by innovations in drug delivery systems and lead to improvement of the efficiency of many new biopharmaceuticals. Designing of novel transdermal delivery systems garnered tremendous attention in both cosmeceutical and pharmaceutical research and industries, which offers a number of advantages. Developing safe and efficient siRNAs delivery vectors is essential for effective treatment of skin diseases. In recent years, significant progress has been made in the creation of delivery systems using lipids, polymers, cell-penetrating peptides, nanoparticles and other biologically active agents. In this review we will focus on the recent advancements in transdermal siRNAs delivery vectors, such as liposomes, dendrimers, cell-penetrating peptides, and spherical nucleic acid nanoparticles.

## 1 Introduction

**Fig. 1:**
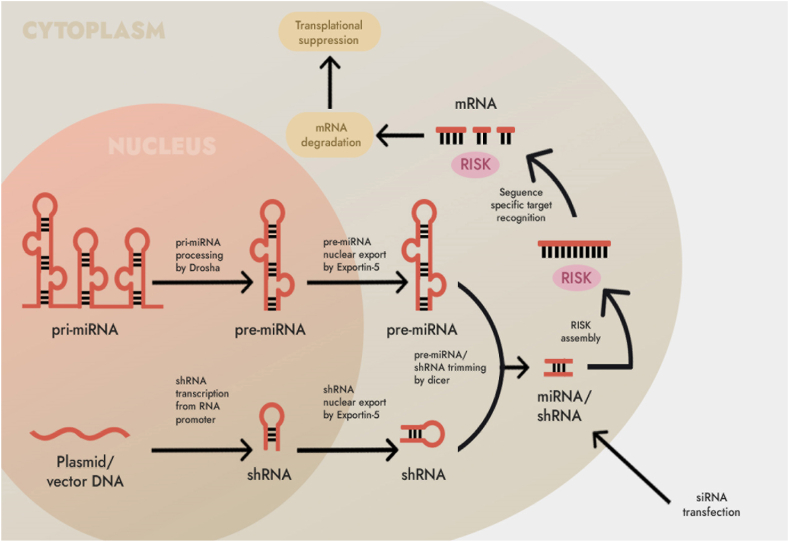
The siRNAs pathways for RNAi in mammals.

RNA interference (RNAi) is a gene expression regulation mechanism that induces post-transcriptional gene silencing in eukaryotic cells [[1], [2], [3], [4], [5]]. SiRNAs is one of the powerful tools of RNAi and has been applied in the treatment of many diseases (Fig. 1).

RNA interference (RNAi) is a highly conserved mechanism for gene silencing that involves the use of double-stranded RNA (dsRNA) to initiate the degradation of targeted mRNA sequences [4]. The design of siRNA duplexes, which are capable of interfering with the expression of specific genes, can be achieved by targeting at least a 20-nucleotide segment of the corresponding mRNA [5,6]. RNAi is extensively utilized in gene silencing and drug development due to its remarkable specificity, potent efficacy, minimal side effects, and straightforward synthesis methods [7,8]. The skin is the most accessible organ for siRNA, and siRNA therapy is especially suitable for the treatment of skin diseases. Many skin diseases, such as psoriasis, pigmented dermatosis, androgenetic alopecia, have already carried out clinical trials of siRNA therapy [7]. However, it is difficult for siRNA to break through the skin barrier and reach target cells to exert interference effects. Therefore, the development of transdermal delivery vehicles is currently a hot spot in the application of siRNA in the treatment of skin diseases. The limited skin penetration of siRNA, antisense ODN, and decoy ODN is primarily attributed to their high ionization, low lipophilicity, and high molecular weight [2]. An intriguing strategy involves the use of decoy molecules to modulate endogenous transcriptional regulation [2]. In earlier studies, Morishita and colleagues discovered the crucial role of the transcription factor nuclear factor-kappaB (NF-κB) in transactivating genes associated with myocardial damage, and they demonstrated that synthetic double-stranded DNA with a high affinity for NF-κB could be introduced in vivo as “decoy” cis elements. These decoys interacted with the transcription factor, effectively counteracting the activation of these genes [3]. Building on this approach, Hashim et al. hypothesized that transfecting NF-κB decoy ODN into the skin could potentially improve the allergic response observed in atopic dermatitis [2,8]. This article will review the latest progress of siRNA transdermal delivery, and provide reference for designing safe and efficient siRNA transdermal delivery vehicles and realizing efficient treatment of skin diseases.

## 2 Challenges to be addressed for successful transdermal delivery of sirna

Barriers to transdermal delivery of siRNA mainly include extracellular and intracellular barriers. The extracellular barrier is mainly hindered by the stratum corneum of the skin. The skin is composed of the epidermis, dermis, subcutaneous tissue and skin appendages, which provide a barrier against the invasion of harmful foreign substances. The stratum corneum, the outermost layer of the epidermis, is composed of terminally differentiated enucleated keratinocytes that form a 10–20 μm thick thin layer through desmosome tight junctions. Cells contain a large number of keratin filaments, and the lipid matrix composed of ceramides, fatty acids and cholesterol between cells is bonded together to form the so-called “brick wall structure” [9]. There are three main pathways for active substances to permeate through the skin: transcellular, intercellular and skin appendages, and the structure of the stratum corneum only allows the penetration of lipophilic molecules with small molecular weight (<500 Da), so the stratum corneum is considered to be the most important part of the skin. The main physical barrier that hinders the entry of foreign objects [10]. However, siRNA has a strong polarity and a molecular weight of about 13 kDa, making it difficult to pass through the skin barrier. Therefore, the stratum corneum has become the primary obstacle for siRNAs transdermal delivery [7]. Intracellular barriers range from the penetration of the cell membrane to the action of the siRNAs on the target mRNA. SiRNAs is negatively charged and has a high molecular weight, making it difficult to pass through cell membranes. Even if siRNAs pass through the cell membrane, naked siRNAs are easily degraded and inactivated by lysosomes or nucleases (Table 1) [11]. One of the major hurdles in siRNA therapy is the inadequate transportation of nucleic acids across cellular membranes, including the skin [12]. This challenge has been associated with the outermost layer of the skin, known as the stratum corneum. To address this issue, Chong et al. conducted a study to investigate the efficacy of solid microneedles in delivering unmodified non-self delivery (non-sd-) lamin A/C siRNA to target human lamin A/C mRNA in vitro [12]. They also examined the delivery of Accell modified sd-siRNA targeting the CBL coding region of transgenic Monster Green™ Fluorescent Protein (hMGFP)/CBL mouse mRNA in vivo [12]. The researchers employed a precise coating technique to load siRNA onto individual microneedles. The lamin A/C siRNA exhibited full activity, as evidenced by a significant reduction in lamin A/C mRNA levels and decreased lamin A/C protein in immortalized human keratinocyte cells (HaCaT) [12]. The approach of using coated steel microneedles is straightforward, and the enhanced loading capacity could prove beneficial in clinical scenarios where a higher dose of therapeutic siRNA is required for human skin tissue [12]. The study demonstrated the successful delivery of siRNA using coated solid microneedles, resulting in the silencing of reporter proteins in vivo [12].

**Table 1:** Advantages and disadvantages of siRNA delivery routes.

Delivery route	Purpose	Cons	Pros
Intrathecal/intraventricular injection	Delivery to central nervous system (CNS)	Low patient compliance, direct toxicity to CNS	Bypass the dense blood-brain barrier, high local concentration, reduce systemic side effects
Inhalation/intranasal/intratracheal administration	Pulmonary delivery	Higher loss of drug in aerosol, low patience compliance, especially for intratracheal administration	High local concentration, reduce systemic side effects
Topical application	Transepithelial absorption (oral, rectal, vaginal mucosa)	The need to bypass thick mucosal layer	Higher patient compliance, non-invasive, high local concentration of siRNA, lower dose needed, reduce systemic side effect
Subcutaneous injection	Systemic delivery	Non-specific, skin toxicity	Broad distribution of siRNA, high localization in liver, avoid RES and renal clearance
Intravitreal injection	Localized delivery	Lower patient compliance, eye irritation	High local concentration, bypass systemic barriers
Intravenous injection	Systemic delivery	Non-specific, higher dose needed, clearance by RES and renal	Broad distribution of siRNA, high localization in liver
Local injection	Localized delivery	Not applicable to all organs and tissues	High localized concentration of siRNA, lower dose needed, reduce systemic side effect

### 2.1 SiRNAs transdermal delivery vehicle

An ideal siRNAs transdermal delivery vehicle should be able to overcome the physical and chemical properties of siRNA, meet the skin penetration conditions, and protect siRNA from the intracellular environment. In recent years, many skin delivery vehicles for siRNA have been developed, including liposomes, dendrimers, cell-penetrating peptides, nanoparticles, etc. The advantages of these vehicles include high delivery efficiency, high biocompatibility, and low immunogenicity and targeting, etc. [13].

### 2.2 Lipid-based siRNA delivery system

Lipid carrier is the most widely used drug delivery system and the earliest transdermal carrier. The delivery efficiency of the lipid system is affected by factors such as particle size, charge, lipid ratio, or composition (Table 2).

**Table 2:** The components of a lipid-based siRNA delivery system.

Component	Description
Lipid Nanoparticles (LNPs)	Nanoscale particles composed of lipids that encapsulate and protect siRNA molecules during delivery
siRNA	Short interfering RNA molecules that target specific genes or transcripts for downregulation or silencing
Cationic Lipids	Lipids with positively charged head groups that interact with negatively charged siRNA for complex formation
Helper Lipids	Lipids that provide stability and structural integrity to the lipid nanoparticles
Polyethylene Glycol (PEG)	A hydrophilic polymer added to the surface of LNPs to improve circulation time and reduce immune response
Targeting Ligands	Molecules (e.g., antibodies, peptides) attached to LNPs to enhance their specificity for target cells
Endosomal Escape Enhancers	Compounds that promote the release of siRNA from endosomes into the cytoplasm, improving its efficacy
Surface Modifications	Chemical modifications on the LNP surface to enhance stability, reduce aggregation, or improve targeting
Formulation Methods	Techniques used to prepare lipid-based siRNA nanoparticles, such as thin-film hydration or microfluidics
In vivo Delivery Strategies	Approaches for administering LNPs to target tissues or organs, including intravenous, intratumoral, etc.
Biological Barriers	Cellular and physiological barriers that LNPs encounter during systemic delivery, e.g., the liver or kidneys
Therapeutic Applications	Disease areas where lipid-based siRNA delivery systems have shown potential for therapeutic interventions

**Fig. 2:**
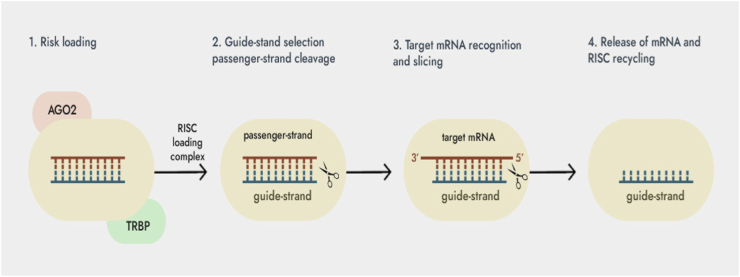
Schematic representation of siRNA silencing mechanism.

The size ranges from 20 nm to 0.5 μm, and the siRNA is mainly delivered to each layer of the skin in the form of encapsulation. In general, this type of lipid system has the advantages of good biocompatibility, low immunogenicity, simple preparation and low development cost due to its similarity with skin lipid components. Popular in the design of transdermal delivery vehicles for siRNAs (Fig. 2).

#### 2.2.1 Liposomes

Liposomes are closed sacs surrounded by a hydrophilic core composed of a lipid bilayer of phospholipids and other lipids. The liposome-mediated siRNAs delivery system wraps siRNA into aqueous vesicles or connects liposomes with siRNAs, and through the deformability of liposomes, the siRNAs carried is squeezed through the stratum corneum cells. However, the structural components of traditional liposomes are similar to the lipid-based components of the stratum corneum and cell membranes. Although their biocompatibility is improved, they are prone to aggregation during the transdermal process and cannot penetrate deep into the skin. At the same time, the phospholipids in the structure are easy to oxidize. However, the stability is affected, so traditional liposome delivery of siRNA is rare in skin application research [14,15]. In order to improve the transdermal delivery of liposomes carrying siRNA to the target, traditional liposomes have been optimized, one of which is the development of siRNA lipid-based delivery systems based on cationic liposomes, such as 1,2-di Octadecenyl-3-trimethylamine-propane (DOTMA), 1,2-dienyl-3-trimethylamine-propane (DOTAP), etc. [16]. A lipoprotein complex delivery system based on cationic lipid carrier DOTAP was used for transdermal delivery of siRNA targeting Kelch-like ECH-associated protein 1 (Keap1), which effectively inhibited the expression of NF-E2-related factor 2 (Nrf-2), and improved wound closure in a mouse model of diabetes [17]. Elastic liposomes are vesicles composed of phospholipids and edge activators (highly mobile surfactants with a high radius of curvature) that exhibit a remarkable ability to penetrate the skin [6]. What sets deformable liposomes apart from conventional liposomes is their exceptional deformability and stress-dependent adaptability, which enables them to squeeze through cellular gaps despite their larger average size. In various research studies, elastic liposomes have gained increasing attention as a delivery system for siRNA. The rationale behind their use lies in the challenges associated with siRNA delivery into the body, primarily hindered by the skin's barrier properties. Additionally, the delivery of “naked” RNA molecules in vivo is challenging due to degradation by endogenous enzymes like ribonucleases [18]. Furthermore, the negative charge, hydrophilic nature, and relatively large size of siRNA (13 kDa) impose limitations on their ability to cross biological membranes [19].

#### 2.2.2 Niosomes

Niosomes are monolayer or bilayer structures self-assembled by nonionic surfactants [20]. Compared with liposomes, liposomes have higher stability and encapsulation efficiency, better flexibility and strong permeability, and lower production costs. Niosomes can reversibly reduce the barrier properties of the stratum corneum, allowing active ingredients to pass through the epidermis faster. Many commercially available skin care products use niosomes to deliver active ingredients. However, the delivery efficiency of niosomes is affected by many factors, such as the nature and structure of surfactants, the properties of encapsulated drugs, and the shape of membrane components. At present, the concept of nanospheres with a core-shell structure has been derived from niosomes, with a size between 10 nm and 200 nm, which can more effectively deliver active ingredients to the deep layer of the skin [21]. Topical application of these vehicles to deliver iRNA may show greater gene silencing capacity than liposomes.

#### 2.2.3 Ultradeformable vesicles (UDV)

UDV, also known as flexible nano-liposome, because of its high deformability and hydration gradient driving force, it is easier to deliver the loaded drug to the epidermis and dermis through the micropores on the skin, which overcomes the disadvantages of traditional liposomes. The disadvantages of stability and poor skin permeability are expected to become a new generation of drug targeting carriers [22]. At present, there are three kinds of UDV that have been paid more attention to: transferosomes, ethosomes and ethanol transfersomes. The biggest difference is that active substances of different polarities are incorporated, so the permeability to the skin is also different [23].

Transfersomes are first-generation UDVs, mainly composed of phospholipids and edge activators (EAs) such as sodium cholate (NaCho), sodium deoxycholate, Span, Tween, and dipotassium glycyrrhizinate [24]. The EAs/phospholipid ratio is a key factor affecting its penetration depth and delivery efficiency in the skin layer. The incorporation of EAs increases the hydration of the stratum corneum and enhances the affinity of the skin. At the same time, EAs can easily change the shape of the vesicles to cope with external mechanical pressure, so that they can squeeze through the channels with a diameter of only one tenth of the vesicles [25,26]. Dorrani et al. found that DOTAP/NaChol 6:1 delivery body can penetrate the skin layer and evenly deposit BRAF-siRNA in the dermis in the study of melanoma treatment [27]. When the preparation ratio is adjusted to 8:1, it can effectively penetrate the stratum corneum and deposit in the lower epidermis/upper dermis, and the delivery rate of siRNA is the highest under this ratio. However, transfersomes formulated at a ratio of 10:1 can only be deposited in the upper epidermis after overcoming the stratum corneum barrier.

Ethosomes are vesicles composed primarily of phospholipids, 20%–45% lower alcohols, and water [28]. Due to the high dose of ethanol, a highly elastic vesicle membrane is formed, which is soft, plastic and strong deformable. At the same time, ethanol bodies can be embedded in the lipid molecular layer of the skin, destroy and dissolve the lipids in the stratum corneum, and improve the delivery of highly hydrophilic molecules such as siRNA to the deep layer of the skin under closed and non-closed conditions [29]. Chen et al. designed a SPACE-penetrating peptide-modified alcohol liposome, and this combination system promoted the penetration and accumulation of GAPDH-siRNA in pig skin [30].

Ethanol transfersome is a new type of flexible lipid carrier developed by Song et al., in 2012, which combines the advantages of ethosome and transferosome. In addition to the basic lipid components, it also has a high content of ethanol (up to 30%) and edge activators, so that its penetration and retention in the skin are stronger than ethosomes and transfer bodies. At the same time, ethanol transfer bodies can Stable encapsulated siRNA for up to 4 weeks [31,32]. In a skin humanized mouse model of psoriasis, DEFB4-siRNA encapsulated in alcohol transporters was accurately delivered to the epidermis to inhibit the expression of target genes and improve the psoriasis-like features on the back of mice [33].

With the continuous optimization of lipid system in the design of transdermal targeted delivery of siRNAs, it may become a promising therapeutic tool for the treatment of skin diseases such as psoriasis. However, affected by size, charge, etc., the lipid system still needs to improve the problem of low encapsulation efficiency.

### 2.3 Polymer-based siRNAs delivery system

Polymer-based delivery vehicles have the advantages of biocompatibility and biodegradability, and have attracted more and more attention in the research of siRNA transdermal delivery vehicles. They not only solve the problem of low siRNA carrying rate, but also show high transfection rate. Most polymers usually contain high-density positive charges, and they interact with negatively charged siRNAs through electrostatic binding to spontaneously form compact siRNA-polymer complexes. It can greatly reduce the negative charge on the surface of siRNA, further improve its stability, and at the same time overcome the obstacle that naked siRNA cannot easily pass through the cell biomembrane. In addition, these cationic polymers with high charge density usually have a “proton sponge effect”, which is beneficial to assist oligonucleotides to escape from endosomes and protect siRNA from degradation, thereby improving siRNA transfection efficiency [34]. Due to the consideration of biodegradability, the current polymer-based siRNA delivery vectors are mainly developed in the direction of biodegradable polymers such as dendritic macromolecules.

#### 2.3.1 Polyethyleneimine (PEI)

PEI is a well-studied polymer carrier for the delivery of nucleic acids. The structure of PEI contains many amino groups and presents a high density of positive charges, which promotes its good complexation and pH buffering capacity in the pH range involved in the endosome/lysosome pathway, protecting nucleic acids from Lysosomal degradation, which is also a key factor for its high transfection efficiency [35,36]. The unique properties of PEI make it widely used as siRNA delivery carrier. However, a significant disadvantage of PEI is the issue of cytotoxicity. PEI exists in two forms, linear and branched. With the increase of molecular weight and branched structure, the transfection efficiency is relatively continuously improved, but the cytotoxicity exhibited is also increased. Therefore, linear or low molecular weight PEI seems to have good transfection efficiency and low toxicity compared with branched PEI. However, low-molecular-weight PEI has fewer sites for siRNA binding and cannot maintain structural stability in an acidic environment, which greatly limits the ability of PEI as a delivery carrier [37,38]. To overcome this shortcoming, various modifications have been made to PEI to optimize PEI delivery vectors without changing its physicochemical properties. For example, in the process of skin scar research, the viability of cells treated with PEI with a molecular weight of 25 kDa was only less than 30%, while PEI modified with sorbitol (PSPEI) significantly reduced cytotoxicity, and the cell viability increased to 80% [39]. Connective tissue growth factor (CTGF) was highly expressed in the wound, CTGF-siRNA was delivered to the wound to silence CTGF, and the scar formation during wound healing was reduced. The wound treated with PSPEI healed earlier than the bare siRNA or 25 kD-PEI group, and wound scars shrink. Furthermore, stearic acid-modified PEI (StA-PEI) delivered siRNA to B16 melanoma cells to achieve highly efficient downregulation of STAT3, and StA-PEI required less content than PEI to complex siRNA, suggesting its potential may be less toxic [40].

In order to mitigate its toxicity, PEI has been conjugated with other polymers such as chitosan, hyaluronic acid (HA), cyclodextrins (CD), and PEG to form nanoparticles (NPs) that can protect siRNAs and facilitate their escape from endosomes.

Chitosan, a biodegradable, biocompatible, and non-toxic polymer, has limited transfection efficiency for siRNAs [41]. However, this limitation can be overcome by coupling chitosan with PEI. For instance, Huh and colleagues developed glycol-chitosan (GC)-PEI-siRNA NPs that demonstrated strong tumor accumulation and target downregulation in vivo [42]. While this study showed promise, the authors did not provide evidence of in vivo anti-tumoral activity since the NPs were designed to carry an siRNA targeting red fluorescent protein (RFP) expressed by xenograft tumors. Another study by Zhang et al. involved the development of a dual-targeting chitosan-PEI nanosystem incorporating the antineoplastic drug Lonidamine. The siRNA targeting the apoptosis inhibitor protein Bcl-2 was covered by a layer of PEG-Poly (acrylic acid)-folic acid to prolong circulation and enhance cancer cell targeting. The chitosan-PEI polymer also incorporated the mitochondria-targeting ligand triphenylphosphine (TPP), enhancing the mitochondrial activity of Lonidamine. While this formulation showed promising results in vitro, further investigation is required to determine its in vivo activity [43].

HA, a polysaccharide found in the extracellular matrix (ECM) and synovial fluids, has been used to target CD44, which is highly expressed in various tumors [18]. Ganesh and colleagues developed HA-PEI/PEG NPs to deliver siRNAs targeting the proteins SSB and PLK1, demonstrating in vivo siRNA activity in xenograft tumor models. However, the knockdown efficiency was highly dependent on tumor vascularization, indicating that tumor accumulation was not solely mediated by the active targeting ligand HA [44].

CDs are cyclic oligosaccharides composed of a macrocyclic ring of glucose subunits joined by α-1,4 glycosidic bonds. β-CD nanosystems have a hydrophobic interior and hydrophilic exterior, making them suitable for enhancing the pharmacokinetic properties of loaded hydrophobic drugs. The cationic polyamine backbone of CD allows for electrostatic interaction with siRNAs, making them ideal candidates for synergistic drug delivery [45]. Wang and colleagues developed a CD-PEI conjugate adsorbed to gold nanorods for the co-delivery of docetaxel (DTX) along with siRNAs targeting the protein p65. The study demonstrated that p65 blockade inhibited the NF-κB pathway and its downstream cascade, enhancing the effect of DTX and reducing tumor growth in vivo. Notably, the nanoplatform facilitated tunable hyperthermia upon irradiation with a near-infrared (NIR) laser, triggering DTX release from the DTX-CD-PEI-siRNA complexes and inducing siRNA endosomal escape. Furthermore, p65 knockdown sensitized 4T1 breast cells to DTX treatment by suppressing the expression of the anti-apoptotic gene, Bcl-2. Treatment of mice bearing 4T1 tumors with DTX-CD-PEI-siRNA nanorods inhibited primary tumor growth and reduced the formation of lung metastasis, suggesting a synergistic effect of NIR irradiation, DTX, and p65 siRNA on tumor cells and the tumor vasculature with significant translational [[46], [47], [48], [49], [50], [51]].

#### 2.3.2 Dendrimer

Dendrimers are nanoscale macromolecules with a dendritic structure [52] (Fig. 2). Different from conventional linear and branched polymers, dendritic polymers have the following advantages: 1) Controllable size and lipophilicity, resulting in spherical morphology of dendrimers and their ability to pass through cell membranes [53]; 2) has abundant end groups that can be modified with different ligands, such as probes, targeting ligands, etc. These modifications reduce the toxicity of siRNA-dendritic polymers during delivery and improve targeting [54]; 3) The excellent flexibility makes the steric hindrance low, which is beneficial to combine with siRNA and form stable nanoparticles of about 70 nm, assisting siRNA to escape from the acidic endosomal environment, thereby promoting the release of siRNA and achieving gene silencing effect [55,56]. At present, a variety of dendrimers that have achieved good results in siRNA delivery have been developed, such as fluorinated dendrimers with high loading capacity, amino acid functionalized dendrimers with excellent biocompatibility, and fluorinated dendrimers with high transfection capacity. Carbosilane dendrimers, in addition to polyamine dendrimers, polypropyleneimine dendrimers, polylysine dendrimers, etc. [6]. In recent years, in order to further improve the transdermal delivery efficiency of dendrimers, many research teams have begun to redesign or optimize them. Liu et al. self-assembled amphiphilic dendrimers into vesicular nanostructures for delivery of siRNA to various cell types, including primary cells and stem cells, showing high delivery efficiency and successful gene silencing [57]. Cyclodextrin-modified polyamide-amine (PAMAM) cationic star polymers successfully delivered MMP9-siRNA to fibroblasts in a skin healing experiment in diabetic rats, leading to downregulation of MMP-9 expression and promoting diabetic wound healing [58]. The gene silencing efficiency of PAMAM modified with C12 saturated alkyl chain can reach 80% when delivering a very low dose of siRNA (10 nmol/L) [59]. Bioreducible fluorinated peptide second-generation dendrimers carrying siRNA-VEGF showed excellent gene silencing efficacy of 65% [60]. However, it is difficult to prepare high-efficiency siRNA-dendritic polymer delivery carriers. High-generation dendritic molecules have a high ability to bind siRNA. Although the delivery efficiency is improved, the steric hindrance is also relatively increased, resulting in Its preparation difficulty increases, and it is difficult to produce on a large scale, which limits its wider application.

#### 2.3.3 Polylactic-co-glycolic acid (PLGA)

PLGA is a biodegradable and safe polymer widely used in drug delivery, including siRNA delivery. At the cellular level, PLGA easily penetrates the cell membrane or facilitates the entry of siRNA into cells through endocytosis, and releases siRNA quickly to the target site before lysosome degradation. However, it has been reported that the encapsulation efficiency, drug release and transfection efficiency of PLGA combined with siRNA alone are limited, which may be affected by the anionic charge and polarity of siRNA. The use of PEI-modified PLGA nanoparticles improves the encapsulation efficiency and release of siRNA, improves the retention of siRNA in the polymer, and promotes the release of siRNA to the cytoplasm [61]. These studies suggest that PLGA nanoparticles are also a potential method for efficient skin delivery of siRNA.

Although some gratifying progress has been made in the research of siRNA-polymer carriers, the toxicity problem is still a major obstacle limiting the clinical transformation of polymer carriers. Most of the polymeric transdermal carriers suitable for siRNA carry more positive charges on the surface, so that they maintain a high transfection efficiency, but too much positive charges will also increase the cytotoxicity of the complex, although it can be passed Surface modification can reduce the toxicity, but the safety issue has always been the focus of people's most attention, and the safety and toxicity evaluation must be carried out before the polymer carrier enters clinical application.

### 2.4 Cell penetrating peptide (CPP)

CPP, as a low-toxic and effective bioactive molecule delivery carrier, has also become a candidate therapeutic tool for transdermal delivery of siRNA. CPP usually consists of a short sequence of no more than 30 amino acids and can be divided into three categories: cationic, amphipathic and hydrophobic.

Bioactive macromolecules are introduced into cells through tissue and cell membranes through energy-dependent or independent mechanisms, and have extremely high transmembrane transport efficiency [62]. Several CPPs, such as TAT, polyarginine, TD-1, SPACE peptide, etc., have been shown to enhance the delivery of siRNA on the skin. TAT (HIV-1 cell-penetrating peptide) is an arginine- and lysine-rich cationic peptide that translocating into cells by overcoming skin barriers in an energy-dependent manner [63]. When the TAT carrier carries siRNA and delivers it to the skin in the form of nanoparticles, TAT-siRNA nanoparticles can pass through the hair follicle when the diameter is larger than 70 nm, and the particles below 70 nm can penetrate through the epidermis [64]. TD-1 (ACSSSPSKHCG) and SPACE peptides promote transport across cellular pathways by binding to keratin in skin keratinocytes [65]. CPP is conjugated to the delivery in a covalent or non-covalent form. The covalent binding is relatively time-consuming and requires high specificity for the delivery. Relatively speaking, non-covalent conjugation is more flexible. Negatively charged siRNA Form a non-covalent complex with CPP through electrostatic interaction, which avoids any chemical modification of siRNA, thereby greatly retaining the activity of siRNA ability [66]. Therefore, many studies have begun to chemically modify CPP or combine it with another siRNA carrier in multimeric form to allow its unhindered uptake and facilitate its release. Such as Tat peptide (GRKKRRQRRRCG) and AT1002 (FCIGRLCG) double peptide system [67,68] to enhance the gene interference efficiency of siRNA in the skin. siRNA is electrostatically complexed with Tat peptide to enhance intracellular delivery, while AT1002 peptide acts as a modulator of skin cell tight junctions to enhance complex penetration into the epidermis and dermis. Intradermal penetration of siRNA was studied using a mouse model of atopic dermatitis [69]. AT1002 and stearic acid (STR)-modified CH2R4H2C peptide were used as delivery carriers for siRNA, and a strong fluorescent signal of siRNA could be detected at 5–10 μm in the epidermis 1 h after application. After 10 h, the siRNA fluorescence signal penetrated deep into the skin at 50 μm; while the unmodified CH2R4H2C peptide or STR-CH2R4H2C peptide carrying siRNA only stayed at 5–10 μm in the epidermis. In addition, many membrane-penetrating peptides also use nanotechnology to optimize and enhance their delivery systems. For example, STP705, an anti-fibrosis siRNA therapeutic candidate drug that is undergoing phase IIa clinical research, uses polypeptide nanoparticles (PNP) to enhance the delivery system and The dual targeted inhibitory properties directly reduced cutaneous wound fibrotic activity and inflammatory activity, reducing cutaneous hypertrophic scar formation [70].

In addition to the delivery advantages of CPP, it is also worth noting that the cell specificity of most primary CPPs is low, resulting in low targeting, and it needs to be optimized for future clinical research applications. In addition, the types of CPPs that can be applied to siRNA transdermal delivery are limited, and efficient and safe CPPs need to be further developed.

#### 2.4.1 PLL-derived nanosystems

PLL, like PEI, is extensively studied for the development of nanocarriers for nucleic acid delivery. Compared to PEI, PLL has better biocompatibility and biodegradability [71]. However, PLL/siRNA and PLL-PEG/siRNA polyplexes are more susceptible to interactions with serum proteins, which can hinder their ability to effectively silence target mRNAs [72]. These interactions indicate that serum albumin and other polyanions can compete with siRNAs for binding to PLL, causing particle instability and siRNA disassembly. Interestingly, the limitations can be mitigated by PLL derivatives that enhance serum stability. For instance, a novel PEG-PCL-PLL polymer, incorporating polycaprolactone (PCL), has been developed to bind siRNAs and form micelles [73]. In vitro studies have shown that this formulation exhibits a silencing efficiency similar to lipofectamine 2000 and superior to PEI. In another study, PEG-PCL-PLL NPs were developed by Xiao et al. incorporating platinum-based chemotherapeutic agents and Bcl-2-targeting siRNA for cancer treatment. In vitro experiments demonstrated significant downregulation of Bcl-2 mRNA levels and enhanced cell death compared to free drugs in multiple cancer cell lines [74]. However, these NPs have not been tested in murine tumor models.

Furthermore, PLL has been conjugated to melanin, a biocompatible pigment known for its excellent photothermal properties. Melanin can generate heat under near-infrared (NIR) irradiation, which can facilitate siRNA endosomal escape. The resulting melanin-PLL polymer loaded with survivin-targeting siRNA exhibited a strong inhibitory effect on 4T1 tumor cell growth both in vitro and in vivo [75]. The development of lung metastases was significantly reduced in treated mice compared to controls. Additionally, Sun et al. developed a pH-responsive triblock polymer composed of poly aspartyl (N-(N′,N'-diisopropylamino ethyl)) (PAD) conjugated to PLL-PEG. This polymer facilitated siRNA and DOX delivery to cancer cells. The NPs loaded with DOX and Bcl-2-targeting siRNA showed tumor accumulation in HepG2/adriamycin (ADM) tumor-bearing mice, resulting in reduced tumor growth and increased survival compared to controls [76]. Wang et al. recently developed a similar nanosystem incorporating a disulfide bridge between PEG and PLL (PEG–SS–PLL) to enable PEG release in endosomes, facilitating siRNA delivery to the cytoplasm of cancer cells. This formulation included a VEGF-targeting siRNA to reduce angiogenesis in the tumor microenvironment (TME). In vivo studies in a HepG2 xenograft murine model demonstrated reduced tumor growth and decreased VEGF expression in the treated group compared to controls [77].

### 2.5 Nanoparticle-based siRNAs delivery system

Various new drug delivery vehicles based on nanoparticles have gained a certain position in the field of transdermal delivery due to their unique advantages [78].

1) Regulation of stratum corneum fluidization according to size, shape, surface charge, and balanced hydrophilic-hydrophobic properties;
2) The nano-scale delivery carrier is in close contact with the skin surface, the residence time of the drug is prolonged, and it has the ability to control the release of the drug;
3) Modified with cell-specific ligands to improve targeting;
4) Nano-carriers can release drugs to the deep layer of the skin through skin appendages such as hair follicles.

Based on these advantages, nanocarriers are developed as the most potential transdermal delivery tool for skin gene therapy.

#### 2.5.1 Lipid-polymer hybrid nanoparticles (LPN)

LPN combines the properties of polymer nanoparticles and liposomes, showing great advantages in terms of structural integrity, storage stability, controlled release ability of the polymer core, biocompatibility and bioavailability, etc., and has rapidly developed into a powerful gene delivery vehicle [79]. LPN is mainly composed of three parts: one is a biodegradable polymer core encapsulating the drug, and the other is an inner lipid layer covering the polymer core [80]. The role of the molecular barrier minimizes the leakage of the encapsulated substance and slows down the degradation of the polymer by limiting the diffusion into water, thereby achieving the delayed release of the encapsulated substance; the third is the outer lipid layer of PEG, which is used to stabilize the carrier, In addition, the incorporation of lipids also increases its ability to penetrate the skin layers. Shi et al. developed lipid hybrid PLGA nanoparticles [81]. Compared with PLGA and PLGA-PEG, the siRNA encapsulation efficiency of hybrid nanoparticles reached 78–82%, while PLGA and PLGA-PEG only encapsulated the required 4%–8%. small RNA. Based on this, Desai et al. developed a novel hybrid lipopolymer nanocarrier (CyLiPns) with a ring-shaped cationic head: efficient delivery of TNFα-siRNA and capsaicin (Cap) to the 360 μm of the dermis demonstrates synergy in the treatment of chronic inflammatory skin diseases [82].

#### 2.5.2 Spherical nucleic acid nanoparticle conjugates (SNA-NCs)

SNA-NCs is a kind of nucleic acid delivery carrier with a spherical nanoparticle as the core, and highly oriented oligonucleotides are densely attached to the surface of the nanoparticle to form a spherical structure with a size between 5 nm and 400 nm [41]. Materials constituting the core structure of SNA-NCs include a variety of inorganic particles (gold, silver, platinum, silica, etc.), organic materials (liposomes, polymers, etc.) or hybrid structures (metal-organic frameworks), these nanoparticle conjugates are essentially inert materials with high stability and strong antibacterial properties, and neither require cationic transfection materials nor additional modifications to drive into cells [42]. Cosmetics giants like L'Oreal and L'Core Paris are using gold nanoparticles to create more effective creams and lotions. So far, SNA-NC has been verified in more than 50 cell lines, tissues and organs for its high-efficiency penetration, targeting, rapid uptake and low immunogenicity. To demonstrate that siRNA-SNA can penetrate skin for gene regulation, Zheng et al. designed gold nanoparticles to deliver siRNA, which crossed almost 100% of keratinocytes and mice after a few hours of topical skin application [43]. Epidermal barrier in models and human skin mimics with up to 100-fold more efficient delivery than commercial transfection agents. In the study of wound healing in diabetic mice, topical application of GM3S-siRNA delivered by SNA could completely heal the wounds of diabetic mice within 12 days [18]. Few traces of SNA could be detected in the lungs, kidneys and other organs of the experimental mice. In the treatment of psoriasis, gold nanoparticles with a diameter of about 12 nm were developed to deliver siRNA, and 90% cell transfection efficiency was achieved in only 24 h, and it showed durable intradermal gene knockdown at picomolar to nanomolar concentrations without showing toxicity [44].

In addition to heavy metal nanoparticles, mesoporous silica nanoparticles (MSNPs) have also been developed for nucleic acid delivery. Different from gold nanoparticles, MSNP has a unique porous structure and high specific surface area, which shows high loading efficiency, excellent biocompatibility and chemical stability, and is also easy to synthesize and modify [45]. Lio et al. loaded siRNAs targeting TGFβR-1 into the mesopores of polylysine-modified (PLL) MSNPs to construct MSNPs-PLL complexes, and as the result, transdermal delivery of siRNA to cutaneous squamous cell carcinoma in xenograft model mice significantly down-regulated the expression of TGFβR-1 and inhibited tumor growth by as much as 2-fold [83]. These results indicated the great potential of SNA-NCs as a transdermal delivery vehicle for siRNAs.

## 3 Conclusion

This article provides an overview of the various carrier systems that have been developed for transdermal delivery of siRNA, including lipids, polymers, membrane-penetrating peptides and nanoparticles. Each of these carrier systems have their own advantages and limitations in terms of siRNA delivery efficiency, biocompatibility, and stability. Lipid carriers, for example, offer low immunogenicity and high flexibility, while polymer carriers offer excellent biodegradability. Despite these advancements, there are still many challenges that need to be addressed in order to make siRNA transdermal delivery a viable option for treating skin diseases. These challenges include limited encapsulation space, high cationic charge, and degradation of siRNA by nucleases among others. Additionally, concerns about heavy metal accumulation and scavenging by gold nanoparticles in the body are also a concern.

To address these obstacles, scientists have started to optimize the siRNA carrier system, such as creating a dual-drug delivery system based on liposomes, or developing composite nanoparticles like LPN [84,85]. These methods have been shown to have a more potent effect in silencing genes in the skin than single-component delivery systems. It is believed that in the future, composite nanocarrier-based siRNA transdermal delivery systems will have greater potential for development and could be a promising solution for locally delivering siRNAs in the skin [[86], [87], [88], [89], [90]].

At present, there are more and more researches on the development of siRNAs transdermal delivery system. With the deepening of research, it is believed that in the near future, more new siRNAs transdermal delivery vehicles will be designed, and the siRNA-based RNAi technology will gradually mature, which will make great contributions to the treatment of skin diseases and other diseases.

## Funding

This work was supported by the Bashkir State Medical University Strategic Academic Leadership Program (PRIORITY-2030).

## Author contributions

Albert Sufianov and Aferin Beilerli conceptualized and designed the study. All authors have participated in the acquisition, analysis and interpretation of the data. Valentin Kudriashov has drafted the manuscript. Tatiana Ilyasova and Bu Wenjie contributed to the critical revisions of the manuscript. Ozal Beylerli supervised the research. All authors agreed on the journal to which the article would be submitted, gave the final approval for the version to be published, and agreed to be accountable for all aspects of the work.

## Declaration of competing interest

The authors declare they have no conflict of interest.
